# A Smartphone app for intensive care unit rotation Orientation

**DOI:** 10.36834/cmej.70650

**Published:** 2020-12-07

**Authors:** Olga Bednarek, Osama Loubani, Samuel Jessula, Samuel Minor

**Affiliations:** 1 Division of General Surgery, Dalhousie University, Nova Scotia, Canada; 2 Department of Critical Care, Dalhousie University, Nova Scotia, Canada

## Implication Statement

The Department of Critical Care at Dalhousie University developed a smartphone app to improve the quality of learner orientation to the intensive care unit (ICU). The app increased satisfaction with orientation and was perceived as useful. It was ranked as the second most valued resource for orientation after other residents. There is potential to improve the experience of learners with this popular technology.

## Énoncé des implications de la recherche

Le département des soins critiques de l'Université Dalhousie a mis au point une application pour téléphones intelligents en vue d'améliorer la qualité de l’accueil des apprenants à l'unité de soins intensifs (USI). L'application a permis d'augmenter la satisfaction par rapport à l’accueil et elle a été jugée utile. Elle a été classée la deuxième ressource d’orientation la plus appréciée après « les autres résidents ». L'expérience des apprenants peut être améliorée à l’aide de cette technologie populaire.

## Introduction

Smartphones are omnipresent within medical practice today, with usage ranging from communication to education.^1^ Over 70% of young Canadian physicians use medical smartphone apps.^2^

Due to complex pathology, ICU rotations are especially challenging for learners. The rotation is often an introduction to new procedures and high-stakes medical decision making.^3^ At Dalhousie University, challenges to providing an effective orientation include accommodating learners with disparate backgrounds and different rotation start dates. We looked to a supplemental smartphone app to address this situation.

## Methods

The app’s development cost $1200 CAD, and required hiring an external developer. The app is called “Halifax ICU” and may be downloaded for free. The Apple App Store charges $99 CAD/year to carry it and Amazon Web Services charges $180 CAD/year for hosting. The app is publicly available and updated weekly, allowing easy access to lecture/call schedules. To protect privacy, only learner initials are used.

Surveys assessing satisfaction with orientation materials were distributed to learners (students/residents/fellows) six months before and after the app’s release. Learners were personally given a paper copy of the survey after one week of the rotation, allowing them time to explore orientation materials. The pre-app survey had four questions: one ranking sufficiency of orientation materials on a 5-point Likert scale, and three multiple-choice questions concerning resources. The post-app survey included two additional questions: Did you try the app? How useful did you find it?

As a project within normal educational requirements aimed at quality improvement, the study was exempt from Research Ethics Board review. There were no concurrent orientation improvement initiatives in the ICU.

## Results

Sixty-six surveys were distributed and completed prior to the app’s launch, and seventy-seven afterwards. 75% of respondents tried the app after its launch. At the time of writing, there were 288 installs (85 Android, 203 Apple). It was used an average of six times per month (range 5-7 times/month), per device. Usage statistics are only from users who chose to share their info with Apple.

**Figure 1:**
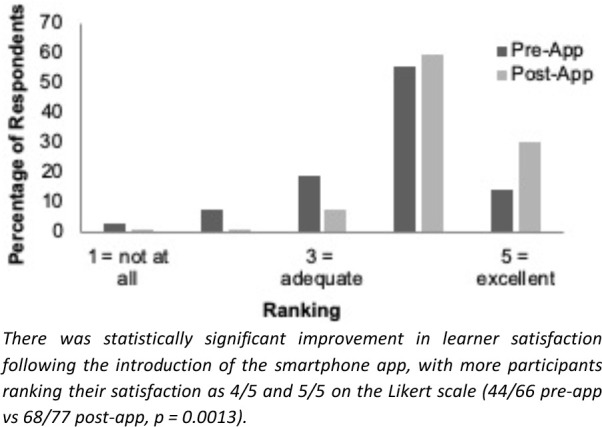
Ranking of learner satisfaction with ICU orientation materials, before and after the launch of the Halifax ICU app.

The app’s usefulness was compared to existing orientation materials, including emails, handouts, and web-based education management software (One45, One45 Software Ltd.), as well as information provided by attending staff, nurses, and other residents. The app was the second-most-cited point of access (after One45), the second-most-cited source for learning about required paperwork (after “other residents”), and the second-most-helpful overall for ICU orientation (after “other residents”). There was a statistically significant improvement in learner satisfaction following the app’s launch (Figure 1).

## Discussion

Effective orientation allows employees to understand their place within organizations, which improves productivity.^4^ This study demonstrates that a smartphone app for rotating residents improved the perceived quality of orientation, which echoes the findings of other studies around medical apps, including trauma services and teaching units.^5,6^ Increase in learner satisfaction may be due to greater variety and accessibility of resources, both through functionality and by demonstrating a department’s commitment to its learners.

As transition to web-based orientation becomes more common in medicine, programs can learn from each other’s experiences in using this technology. Future research could explore cost-effectiveness and how improved orientation affects patient care outcomes (length of stay, etc.). The app itself could also serve as a research tool, facilitating surveys or processing other information for ICU research.

## Conclusion

An ICU orientation app resulted in increased learner satisfaction. It was considered a valuable resource to learn about required tasks.
